# Sedentary behavior and self-harm in adolescents with asthma, rhinitis and eczema

**DOI:** 10.1016/j.jped.2024.08.003

**Published:** 2024-09-05

**Authors:** Mércia L. Medeiros, Auxiliadora D.P.V. da Costa, Ciane J.G. Vieira, Pedro H.N. Silva, Diego A.M. Santos, Maria Sylvia S. Vitalle

**Affiliations:** a Universidade Federal de São Paulo (UNIFESP), Ciências Aplicadas à Pediatria, São Paulo, SP, Brazil; b Universidade Federal de Alagoas (UFAL), Faculdade de Medicina, Maceió, AL, Brazil; c Universidade Federal de Alagoas (UFAL), Instituto de Educação Física e Esporte, Maceió, AL, Brazil; d Universidade de São Paulo (USP), São Paulo, SP, Brazil; e Universidade Federal de São Paulo (UNIFESP), Escola Paulista de Medicina (EPM), Departamento de Pediatria, São Paulo, SP, Brazil

**Keywords:** Adolescents, Asthma, Rhinitis, Eczema, Self-injurious behavior, Sedentary behavior

## Abstract

**Objective:**

To investigate the association between allergic diseases and the tendency to self-harm in adolescents, considering the role of sedentary behavior.

**Methods:**

This was a population-based cross-sectional study, carried out in 2022, with 727 adolescents aged 12 to 19 years, from a capital in the Northeast of Brazil. The authors evaluated the association between each allergic disease (asthma, rhinitis and eczema) and self-harm, sedentary behavior and other variables. The authors performed an adjusted analysis of the associations between each allergy disease and the tendency to self-harm and then adjusted to the presence of family members and sedentary behavior.

**Results:**

The prevalence of asthma, rhinitis and eczema were 18.76%, 36.21% and 12.86%, respectively. Sedentary behavior and tendency to self-harm were more frequently reported in asthmatics (PR 2.16; 95% CI: 1.55 – 3.00 and PR 1.98; 95% CI: 1.47 – 2.68, for sedentary behavior and self-harm respectively), rhinitis (PR 1.53; 95% CI: 1.25 – 1.88 and PR 1.33; 95% CI: 1.09 – 1.62, respectively) and eczema (PR 2.35; 95% CI: 1.54 – 3.58 and PR 1.55; 95% CI: 1.05 – 2.28, respectively). There was a reduction in the strength of this association in the three conditions, which included a loss of association.

**Conclusion:**

High rates of sedentary behavior and self-harm in those with asthma, rhinitis and eczema. Physical activity attenuated the risk for self-harm. It warns about the urgency in detecting these factors, whether in the diagnosis or in the implementation of therapy, seeking to reduce their harmful consequences in the short and long term.

## Introduction

Adolescence, the period between childhood and adulthood, is marked by several changes, including rapid physical, cognitive, social and emotional maturation.^1^ Although genetics constitute one determinant, the environment has also been identified as an important influence on manifestations at this stage, in relation to behavior and vulnerability to risky habits, such as sedentary behavior and self-harm, for example.

Sedentary behavior is understood as a set of activities in which the individual spends most of their time in a sitting position, and is an important public health concern since it has been associated with the early emergence of chronic diseases, such as dyslipidemia, hypertension, diabetes and obesity, and mental disorders, such as depression and anxiety.^2^ WHO (World Health Organization) data reveal that up to 81% of adolescents do not exercise in line with recommendations.^2^ Common activities that are currently seen in adolescent routines, such as the use of mobile devices, computers, tablets, video games and television, have contributed to this increase in sedentary behavior and to lower levels of physical activity.^3^ Particularly following the COVID-19 pandemic, studies show that the amount of time adolescents spend sitting down has increased by approximately 159.5 ± 142.6 min per day.^4^

Particularly in relation to the association with mental disorders, sedentary behavior and time spent sitting down seem to be associated with symptoms of depression, depressed mood and anxiety among children and adolescents, with sedentary behavior being a probable modifiable risk factor for depression.^5^^,^^6^ Anxiety and impulsivity disorders can appear in early adolescence along with risky behaviors such as self-harm, which is a form of psychopathological externalization of anguish and pain aimed at relieving emotional pain, may become manifest. More common in girls, self-harm is a risky behavior with varying degrees of severity, which may even culminate in suicide attempts.^7^

The negative impacts of allergic diseases on general adolescent performance and health can culminate in a higher risk of psychological damage, especially in the case of chronic conditions which, depending on their severity, can affect self-esteem and quality of life.^8^ On the other hand, emotional stress itself affects the manifestation of disease symptoms, propagating a vicious cycle of increased clinical severity and poorer mental health.

However, few studies ^9^ examine the association between allergic diseases and manifestations of, or tendency to, self-harm in adolescents. This study aimed to investigate the association of self-harm with asthma, rhinitis and eczema, considering sedentary behavior as a possible influence.

## Methods

### Participants and study design

This is a population-based cross-sectional study, conducted during the second school semester of 2022, with 727 students aged from 12 to 19, resident in all of Maceio's districts. To ascertain the proportion of public versus private schools, the authors consulted the ratio of enrolled students in the school census, based on lists provided by the Municipal Department of Education (SEMED). The authors then randomly selected schools based on a proportion of 80.6% public and 19.4% private schools. Adolescents with serious cardiorespiratory (as congenital and acquired cardiomyopathies with hemodynamic repercussions, cystic fibrosis, bronchiectasis, pulmonary fibrosis, for example) and dermatological diseases (such as psoriasis, lupus, leprosy, for example) were excluded.

The self-administered questionnaires were distributed in the classroom among adolescents, and answered anonymously to ensure the privacy and reliability of the responses. Conversely, the school was assured access to specialist psychological counseling services on demand from the institution.

### Instruments and the definition of variables

The authors applied standardized and validated, self-completion written questionnaires (QE) to both children and adolescents, to obtain information about sociodemographic data; prevalence of allergic diseases (asthma, rhinitis and eczema); common mental disorders; tendency to self-harm; and sedentary behavior; in addition to complementary semi-structured questions, related to the instruments.

1. Identification of asthma, allergic rhinitis and atopic eczema: using the International Study of Asthma and Allergies in Childhood – ISAAC.^10^
2. Patient Health Questionnaire-9 - PHQ-9 ^11^ for adolescents, to identify the presence of a mental disorder; the authors also posed the question “Over the last two weeks, how often have you been bothered by: thoughts about hurting yourself in some way, or that you would be better off dead?” to determine the tendency to self-harm.
3. Other variables: - Familial allergy: in first-degree relatives, defined by the presence of at least one first-degree relative with asthma, rhinitis, or atopic eczema. Socioeconomic stratification: using the Socioeconomic questionnaire from the Brazilian Association of Population Studies (ABEP),^12^ in line with Brazilian Economic Classification Criteria, which categorizes economic classes into A, B, C, D and E. For the purpose of analysis, the classes were recategorized for dichotomization into 1) A/B/C and 2) D/E. Household smoking: presence of a person living in the house who smokes. - Screen time: total daily time spent using screens (computers, tablets, smartphones, television or equivalent). Dichotomized into less than 2 h and greater than or equal to 2 h. - Gender, type of school (public or private), mother's, father's or legal guardian's education, in this order of priority (more or less than higher education).

### Statistical analysis

The data were tabulated in a Microsoft Excel® spreadsheet and, after coding, were entered into the statistical package Stata version 13.0 (StataCorp, CollegeStation, TX, USA). The variables were analyzed using Pearson's chi-square test, with the associations between asthma, rhinitis and eczema (dependent variables) and the categorical independent variables of interest, as well as between each allergic disease and self-harm, sedentary behavior and the other variables. The authors obtained an adjusted analysis of the associations between each allergic disease and the tendency to self-harm, and then adjusted for the presence of family allergy and sedentary behavior, in order to observe these variables’ interference on the association between asthma, rhinitis and eczema, and a tendency to self-harm.

### Ethical issues

The study was approved by the FAMED-UFAL Research Ethics Committee under registration number 59311222.7.0000.5013; all the participants signed an Informed Consent Form, when applicable**.**

## Results

The authors considered 727 of the 823 adolescents for analysis, that is, those who presented complete data. The prevalence of asthma, rhinitis and eczema were 18.76%, 36.21% and 12.86%, respectively. Almost half the adolescents described sedentary behavior and approximately 1/3 were prone to self-harm. More than 80% exceeded the maximum daily screen time (Table 1) recommended by the Brazilian Society of Pediatrics (SBP).^13^ Sedentary behavior was more frequent in girls (66.95% × 33.04%; PR: 1.85; 95%CI: 1.56 – 2.20), as was the tendency to self-harm (71.49% × 28.51%; PR: 2.29; 95% CI: 1.80 – 2.92).

**Table 1:** Characterization of the sample according to sociodemographic variables, allergic diseases and adolescent screen time, in Maceio, the state capital of Alagoas, 2022 (*n* = 727).

VARIABLES	N	(%)
Gender, Feminine	371	52,25%
Public school	583	80,19%
ABEP classification, D/E class	189	26,07%
Parents' education level, < High school graduate	480	70,59%
Smoking at home	381	53,06%
Family allergy history	250	35,77%
Screentime ≥ 2 h/day	483	85,64%
Tendency to self-harm	246	33,84%
Sedentary behavior	351	48,28%
Asthma	133	18,76%
Allergic Rhinitis	252	36,21%
Eczema	89	12,86%

History of familial allergy in first-degree relatives, as well as sedentary behavior and tendency to self-harm, were more frequently reported in adolescents with asthma, rhinitis and eczema. Allergic rhinitis was less prevalent among adolescents in public schools (Table 2). Although adolescents with asthma and rhinitis spent more time on their screens, this trend was not statistically significant.

**Table 2:** Association between allergic diseases, tendency to self-harm, sedentary behavior and sociodemographic factors.

VARIABLES	ASTHMA				ALLERGIC RHINITIS				ECZEMA			
	n (%)	PR (CRUDE)	CI (95%)	P	n (%)	PR (CRUDE)	CI (95%)	P	n (%)	PR (CRUDE)	CI (95%)	P
Gender, Feminine	76 (20,88%)	1,34	0,97 – 1,85	0,07	140 (38,78%)	1,2	0,97 – 1,47	0,08	52 (14,65%)	1,52	1,00 – 2,30	0,05
Gender, Masculine	51 (15,55%)				103 (32,39%)				31 (9,66%)			
Public school	103 (18,23%)	0,87	0,61 - 1,26	0,47	183 (33,03%)	0,68	0,55 – 0,83	<0,01*	73 (13,25%)	0,86	0,51 – 1,42	0,55
Private school	30 (20,83%)				69 (48,59%)				16 (11,35%)			
ABEP classification, D/E class	35 (19,13%)	1,02	0,72 – 1,45	0,89	55 (30,73%)	0,8	0,63 – 1,03	0,08	21 (11,73%)	0,88	0,56 – 1,39	0,59
ABEP classification, A/B/C class	98 (18,67%)				197 (38,25%)				68 (13,31%)			
Parents' education level, < High school graduate	34 (17,44%)	0,92	0,64 – 1,31	0,63	63 (32,81%)	0,85	0,68 – 1,08	0,18	29 (15,10%)	1,23	0,81 – 1,86	0,33
Parents' education level, > High school graduate	89 (19,02%)				176 (38,43%)				56 (12,31%)			
Smoking at home	75 (20,11%)	1,16	0,85 – 1,59	0,34	130 (35,42%)	0,97	0,80 – 1,19	0,78	55 (15,15%)	1,44	0,97 – 2,15	0,07
No smoking at home	57 (17,27%)				118 (36,42%)				34 (10,49%)			
Family allergy history	59 (24,08%)	1,54	1,13 – 2,10	0,01*	112 (47,06%)	1,57	1,29 – 1,91	<0,01*	45 (18,83%)	1,92	1,30 – 2,82	<0,01*
No family allergy history	69 (15,61%)				133 (29,95%)				43 (9,82%)			
Screentime ≥ 2 h/day	92 (19,45%)	1,56	0,85 – 2,86	0,15	174 (37,74%)	1,38	0,94 – 2,03	0,1	56 (12,25%)	1,18	0,58 – 2,38	0,64
Screentime < 2 h/day	10 (12,50%)				21 (27,27%)				8 (10,39%)			
Tendency to self-harm	67 (27,92%)	1,98	1,47 – 2,68	<0,01*	102 (43,40%)	1,33	1,09 – 1,62	<0,01*	39 (16,81%)	1,55	1,05 – 2,28	0,03*
No tendency to self-harm	66 (14,07%)				150 (32,54%)				50 (10,87%)			
Sedentary behavior	89 (25,95%)	2,16	1,55 – 3,00	<0,01*	148 (44,18%)	1,53	1,25 – 1,88	<0,01*	61 (18,32%)	2,35	1,54 – 3,58	<0,01*
No sedentary behavior	44 (12,02%)				104 (28,81%)				28 (7,80%)			

⁎ Statistically significant associations (*p* < 0,05). PR, prevalence ratio; CI, confidence interval.

As additional data, a greater frequency of sedentary behavior was also observed among adolescents with a tendency to self-harm (82.93%; *n* = 204), compared to the group that did not report these types of thoughts (17.07%; *n* = 42), with a PR of 2.71 (95% CI: 2.34 – 3.14).

When the authors tested for the interference of sedentary behavior on the association between each allergic disease and the presence of a tendency to self-harm, we observed that, after adjusting for family allergy history, there was a reduction in the strength of this association for the three conditions: asthma, rhinitis and eczema, which included a loss of association (Table 3).

**Table 3:** Association between allergic diseases and tendency to self-harm, taking into account the effect of family allergy history and sedentary behavior.

VARIABLES	Model 1 (Tendency to self-harm)	Model 2 (Tendency to self-harm + History of family allergy)	Model 3 (Tendency to self-harm + History of family allergy + Sedentary behavior)
**Asthma**	1,98 (1,47 – 2,68); *p* < 0,01	1,84 (1,33 – 2,54); *p* < 0,01	1,43 (0,99 – 2,05); *p* = 0,05
**Allergic Rhinitis**	1,33 (1,09 – 1,62); *p* < 0,01	1,26 (1,03 – 1,54); *p* = 0,02	1,07 (0,85 – 1,34); *p* = 0,57
**Eczema**	1,55 (1,05 – 2,28); *p* = 0,03	1,41 (0,94 – 2,11); *p* = 0,09	0,99 (0,64 – 1,53); *p* = 0,97

## Discussion

One-third of the adolescents in this study demonstrated a tendency to self-harm, which was more frequently found among girls and those with asthma, rhinitis and/or eczema; this risk is twice as high as that reported in previous studies.^14^

However, although the associations between asthma, rhinitis and eczema, and mental disorders, notably anxiety and depression, have been investigated,^15^ a gap remains, specifically in relation to self-harm behaviors. In adolescence, self-harm can be perceived or suspected, based on signs that involve the incidence of minor traumas, a range of self-mutilations, such as cuts to the skin, scratches, burns, as well as exposure to risky circumstances, which may even culminate in a tendency for, or actual, suicidal acts. With varying levels of severity, this is characterized by a psychopathological externalization of anguish and pain which, although not expressed verbally, is manifested on one's own body, leaving visible and invisible marks.^7^

In chronic diseases common in childhood and adolescence, such as asthma, severe rhinitis and eczema, which have significant impacts on quality of life, self-esteem and psychological damage, the importance of early identification of this type of risk behavior is even more pressing. In a vicious cycle of chronic illness – mental damage – worsening manifestations of physical illness, risk behaviors such as self-harm, symptoms of depression, anxiety and stress are linked to more severe effects on asthma, for example, and can affect self-control, poorer self-management of the disease, poorer control of triggers and interference in health care, prevention and the management of symptoms.^16^

In the period during and following the COVID-19 pandemic, these relationships between risk behavior, mental disorders and chronic diseases were exacerbated, with consequences that have yet to be well defined. Face-to-face contact, touch, exchange, meetings and communication were quickly replaced with virtual contact, due to the high level of health risks and government containment measures, which greatly affected physical and mental health, and had an emotional impact on a large part of the population.^17^ Home confinement encouraged inappropriate behavior in all age groups. Adolescents, that is, individuals exposed to various stressors, both internal and external, faced the greatest challenges in the pandemic, due to academic pressure to maintain high quality, intellectual commitment, and previous levels of productivity.

Adolescents reflected behaviors immediately post-pandemic, as these data were collected in 2022, consistent with other national studies, in which social distancing related to the pandemic promoted significant changes in the lifestyle of children and adolescents, increasing screen time, reducing activity physical activity and worsening the quality of food and sleep.^18^ Other studies have also identified an association between low levels of physical activity and prolonged use of screen time with increased risk of depression, anxiety and self-harm behavior, with the risk being highest among adolescent boys.^19^

This context further reinforced the maintenance of sedentary behaviors in the school environment, given that studies of both groups, asthmatics and controls, undertaken prior to the pandemic, had already revealed sedentary behavior.^20^

In the present study, the authors identified rates for sedentary behavior that were 1.5 to 2 times as high among adolescents with allergic diseases and among girls. Our finding of more frequent sedentary behavior in girls is in line with data in the literature.^21^ The authors know that guidelines about physical activity must be adapted to individual physical condition, age and gender, taking account of sociocultural determinants and the adolescent's own preferences.^22^ Differences in exercising and sedentary behavior between genders can be explained by sociocultural differences, given that from childhood, boys are encouraged to play games that involve sports and more strenuous physical effort, while girls are encouraged to perform lower-intensity practices, linked to the domestic environment.^23^

In the specific case of allergic diseases, adolescents with asthma and rhinitis who stop exercising due to shortness of breath, often experience a worsening of muscular conditioning, limiting their ability to exercise, resulting in more shortness of breath, since this decrease in performance requires the individual to ventilate more in order to maintain this exercise.^24^ In this sense, systematic reviews reveal positive findings regarding regular exercise in asthmatic adolescents and children, leading to improved cardiovascular function and few effects on bronchial hyperreactivity.^25^ Exercising regularly is related to a number of health benefits, such as improved cardiorespiratory fitness, body composition, and cardiometabolic profile.^2^

According to this study asthma, rhinitis and eczema are relatively common diseases in adolescence, with frequencies of 18.76%, 36.21% and 12.86%, respectively, and which are on the rise. The increasing number of allergic respiratory diseases in adolescents is related to a combination of environmental and genetic factors, such as air pollution, frequent contact with synthetic materials, changes in diet, and level of physical activity.^23^ Higher sitting time to study and short sleep time were associated with asthma, allergic rhinitis, and atopic dermatitis in a Korean study. The associations between obesity and these allergic diseases were inconsistent after adjustment for other factors.^26^

Compared to asthma, rhinitis is less severe, but, depending on the frequency and intensity of symptoms, it can also cause significant limitations in daily activities and quality of life with repercussions on both psychological status, physical activity and behavioral risk. sedentary. Atopic eczema, on the other hand, in addition to limiting daily activities due to itching, which can be intense, also induces the stigma of skin lesions, which affect body self-image, self-esteem, and quality of life, also being a risk for behavioral risks such as sedentary lifestyle and self-harm.

Finally, the authors also found that sedentary behavior, which is common in adolescents at risk of self-harm (82.93% of cases), can modify and attenuate the association between this behavior and allergic diseases. The authors did not find evidence of this effect modification for this association in the literature. The role of an unhealthy lifestyle in suicidal behaviors has become a matter of concern and growing interest,^27^ studies show that more leisure time being sedentary is associated with higher odds of suicide attempts.^28^ This observation is important because changes in lifestyle habits and sedentary behavior are modifiable factors that can affect mental health since active behavior is beneficial for improving cognitive function, depression and self-esteem.^29^ In fact, sedentary behavior may have been confounding in the association between allergic diseases and self-harm, since it was associated with both. Confounding is a bias because it can result in a distortion in the measure of association between an exposure and a health outcome. This was a limitation of our findings.

Another study limitation is that, since it involves cross-sectional research, a causal relationship cannot be established between allergic diseases, sedentary behaviors, and self-harm. Further, assessment through questionnaires depends on possible changes in mood and on the adolescent's willingness to reveal the information requested, particularly in relation to self-harm. The delivery of questionnaires for self-completion results in losses and may have contributed to a return rate of around 88.3% (727 / 823).

Other research has found that low physical activity/high screen time subgroups, who did not meet WHO recommendations for PA and screen time, had significantly more depression, anxiety and self-harm behaviors, highlighting the potential role of the interaction between PA and screens in preventing depression, anxiety and self-harm behaviors in adolescents of both sexes.^30^

Similar to what the authors did in the collection in schools, the study opens up the possibility of identifying risk behaviors such as sedentary lifestyle and self-harm, in routine consultations, of adolescents with chronic conditions, using a simplified instrument, low-cost and quick-to-apply, very useful in primary care. The identification of the risk of self-harm should be referred for specialized psychological care, as an early intervention that can block the progression to suicidal thoughts and ideation or even a suicide attempt, for example. On the other hand, sedentary behavior is a modifiable factor, which can also be identified and subject to intervention, including having an effect on the risk of self-harm in these adolescents. In particular, in a scenario of increasing frequency of allergic diseases and following a pandemic, in which an increase in both sedentary behavior and mental disorders has been observed, early identification and intervention should be a priority.

Therefore, it is essential that health professionals are aware of these factors and include the assessment of physical activity and mental health when monitoring allergic patients. Promoting healthy habits, encouraging physical exercise and psychological support can be important measures to minimize the negative impact of sedentary behavior and mental disorders on the health of allergic patients.

## Conflicts of interest

The authors declare no conflicts of interest.
